# Identification and validation of an H2AZ1-based index model: a novel prognostic tool for hepatocellular carcinoma

**DOI:** 10.18632/aging.205497

**Published:** 2024-02-01

**Authors:** Jiamin Gao, Qinchen Lu, Jialing Zhong, Zhijian Li, Lixin Pan, Chao Feng, Shaomei Tang, Xi Wang, Yuting Tao, Xianguo Zhou, Qiuyan Wang

**Affiliations:** 1 Laboratory of Infectious Disease, Nanning Infectious Disease Hospital Affiliated to Guangxi Medical University and The Fourth People’s Hospital of Nanning, Nanning, China; 2 Center for Genomic and Personalized Medicine, Guangxi Medical University, Nanning, China; 3 Guangxi Key Laboratory for Genomic and Personalized Medicine, Guangxi Collaborative Innovation Center for Genomic and Personalized Medicine, Nanning, China; 4 Department of Clinical Laboratory, The First Affiliated Hospital of Guangxi Medical University, Nanning, China; 5 Department of Blood Transfusion, Guangxi Medical University Cancer Hospital, Nanning, China

**Keywords:** H2AZ1, hepatocellular carcinoma, ubiquitination, WGCNA, diagnostic model

## Abstract

The H2A.Z variant histone 1 (H2AZ1) is aberrantly expressed in various tumors, correlating with an unfavorable prognosis. However, its role in hepatocellular carcinoma (HCC) remains unclear. We aimed to elucidate the pathways affected by H2AZ1 and identify promising therapeutic targets for HCC. Following bioinformatic analysis of gene expression and clinical data from The Cancer Genome Atlas and Gene Expression Omnibus database, we found 6,344 dysregulated genes related to H2AZ1 overexpression in HCC tissues (P < 0.05). We performed weighted gene co-expression network analysis to identify the gene module most related to H2AZ1. The H2AZ1-based index was further developed using Cox regression analysis, which revealed that the poor prognosis in the high H2AZ1-based index group could be attributed to elevated tumor stemness (P < 0.05). Moreover, the clinical model showed good prognostic potential (AUC > 0.7). We found that H2AZ1 knockdown led to reduced superoxide dismutase (SOD) activity, elevated malondialdehyde (MDA) levels, and increased apoptosis rate in tumor cells (P < 0.001). Thus, we developed an H2AZ1-based index model with the potential to predict the prognosis of patients with HCC. Our findings provide initial evidence that H2AZ1 overexpression plays a pivotal role in HCC initiation and progression.

## INTRODUCTION

Primary liver cancer (PLC) is a globally prevalent malignancy with a high mortality rate [1]. Hepatocellular carcinoma (HCC) is the main histological subtype of primary liver cancer, accounting for 75%-85% of cases. HCC has multiple risk factors, including HBV or HCV infection and alcoholic cirrhosis [2]. The primary treatment options for HCC include surgery (hepatectomy), interventional therapy, microwave radiofrequency ablation, targeted therapy, radiotherapy, and chemotherapy. However, most patients are diagnosed with advanced-stage HCC and cannot be curatively treated with surgical resection, *in situ* liver transplantation, or local percutaneous tumor ablation [3]. The treatment options for patients with advanced HCC include radiofrequency ablation (RFA) [4] and transcatheter arterial chemoembolization (TACE) [5]; however, the therapeutic effects are unsatisfactory. Sorafenib is the main targeted drug for treating patients with advanced HCC; however, less than one-third of patients benefit from it and most of them develop drug resistance within 6 months of starting treatment [6, 7]. Therefore, identifying novel HCC prognostic markers and potential drug targets is necessary to help physicians determine the progression of the disease and facilitate the development of potential therapies for HCC.

Genomic and epigenetic changes are important drivers of cancer development [8]. Moreover, histone variants and their post-translational modifications play a crucial role in cancer initiation and progression [9, 10]. Histones, as major chromatin components [11], with their post-translational modifications, are associated with tumor metastasis [12]. Among these, H2A.Z variant histone 1 (H2AZ1, also known as H2AFZ) is present in almost all organisms. It is involved in several physiological processes, such as transcriptional control, DNA repair, and regulation of mitotic heterochromatin [13]. H2AZ1 is the most highly expressed histone variant within the H2A family [14] and is overexpressed in various cancers, including prostate, bladder, non-small cell lung, breast, and colorectal cancers [15–19]. H2AZ1 overexpression in tumors is associated with a poor prognosis [20, 21], suggesting its important role in tumor development and progression. H2AZ1 overexpression is more pronounced in metastatic cancer [22]. Notably, preliminary observations suggest that the abnormal expression of H2AZ plays a significant role in the progression of liver cancer [23, 24], although the molecular pathways associated with H2AZ have not been elucidated. We aimed to further characterize the biological characteristics and affected pathways of H2AZ1 in HCC, thereby providing new and promising therapeutic targets for HCC.

## RESULTS

### Gene co-expression modules characterize the global regulatory pattern of H2AZ1 in HCC

**Figure 1:**
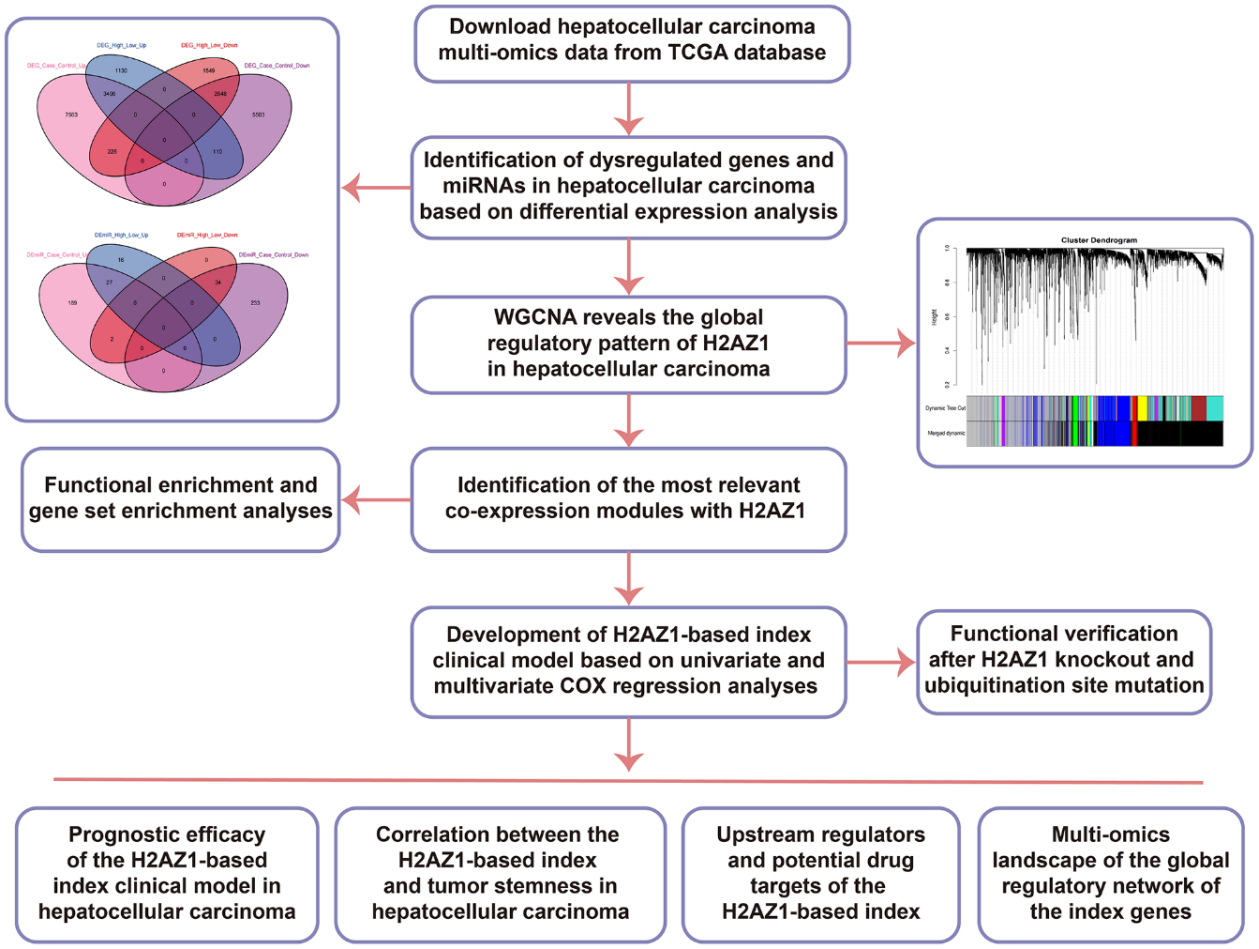
**Flow chart of this study.** TCGA, The Cancer Genome Atlas; DEG: Differentially Expressed Gene; DEmiR: Differentially Expressed miRNA; WGCNA, Weighted Gene Co-Expression Network Analysis; WT, wild type; MUT, mutant.

**Figure 2:**
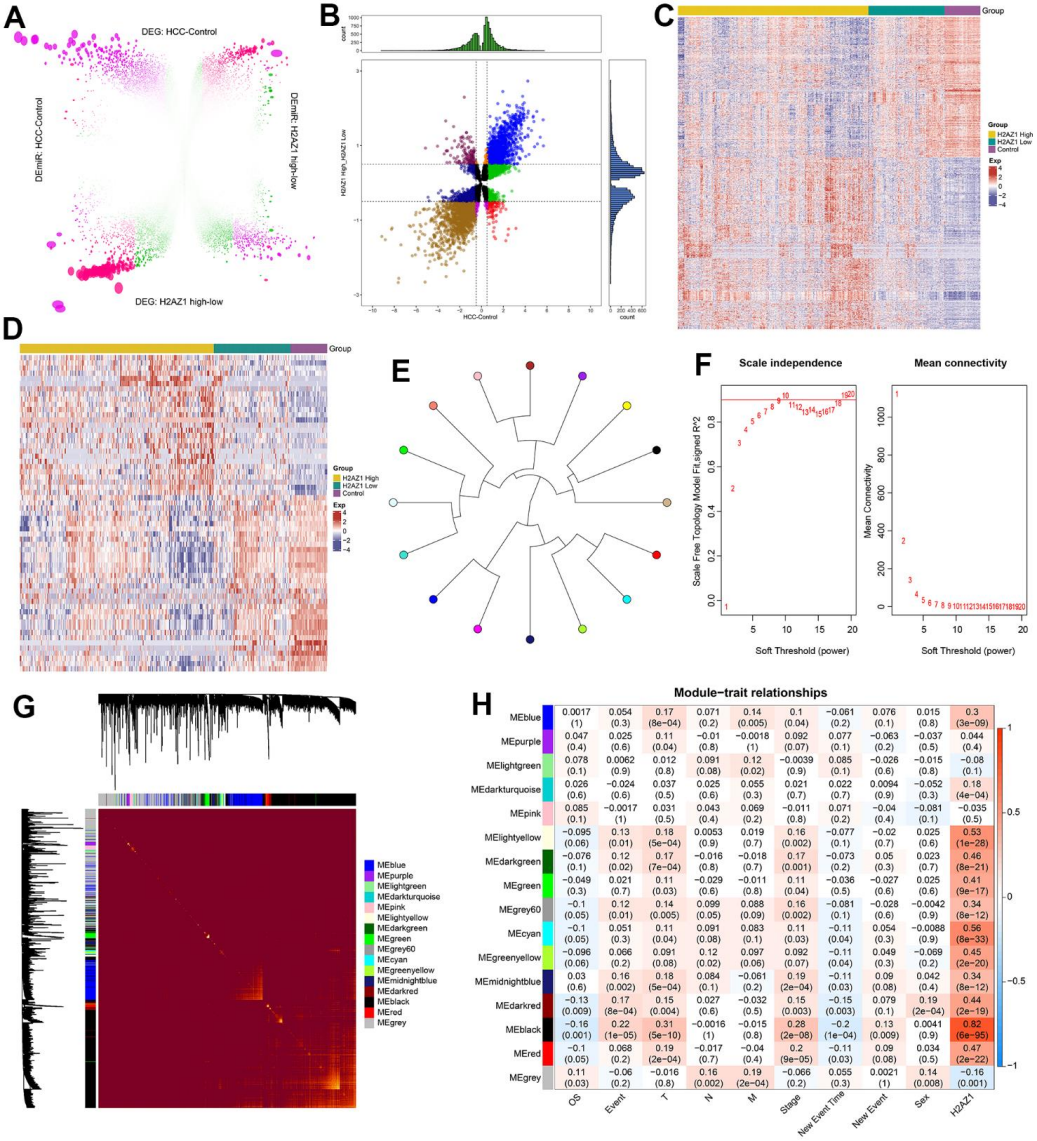
**Weighted gene co-expression network analysis reveals the regulatory pattern of H2AZ1 in hepatocellular carcinoma.** (**A**) Rosette volcano plot showing differentially expressed genes (DEGs) and differentially expressed miRNAs (DEmiRs) in control and hepatocellular carcinoma (HCC), H2AZ1 overexpressed and H2AZ1 underexpressed, respectively (TCGA-LIHC). (**B**) Scatter plot showing differentially expressed genes and miRNAs in HCC affected by H2AZ1. (**C**) Heat map showing the expression of dysregulated genes associated with H2AZ1 overexpression in HCC in Control-H2AZ1 high and low expression groups. (**D**) Heat map showing the expression of H2AZ1-related HCC DEmiRs in the high and low expression groups of Control-H2AZ1. (**E**) Module ring tree diagram showing the adjacency relationship between the co-expression modules of HCC-related dysregulated genes. Gene clustering is represented by different colors. (**F**) Scale independence and mean connectivity analysis for various soft threshold powers. (**G**) Heat map of the module clustering tree showing the gene members of the co-expression module of the H2AZ1 gene in the regulation of HCC. (**H**) Module-trait relationship showing the correlation of gene co-expression modules with H2AZ1 and tumor size (T), lymph node involvement (N), distant metastasis (M), overall survival (OS), age, and gender.

The workflow of this study is illustrated in Figure 1. Through a differential expression analysis of 374 HCC and 50 control samples, we identified 19,746 differentially expressed genes (DEG), comprising 11,285 upregulated DEGs and 8,461 downregulated DEGs, and 485 differentially expressed miRNAs (DEmiRs) consisting of 218 upregulated DEmiRs and 267 downregulated DEmiRs (adjusted P<0.05). Moreover, 9,359 DEGs (4736 upregulated DEGs and 4623 downregulated DEGs) and 88 DEmiRs (43 upregulated DEmiRs and 45 downregulated DEmiRs) were identified between H2AZ1 high and low samples (Figure 2A). In addition, we identified DEGs and miRNAs whose expression levels were consistently up- or downregulated in both sets of differential results, identifying them as the dysregulated genes and miRNAs associated with H2AZ1 overexpression in HCC (Figure 2B). Heatmaps showed significant differences in the expression of these genes and miRNAs between the H2AZ1 high expression group and the H2AZ1 low expression and control groups (Figure 2C, 2D).

Using weighted correlation network analysis (WGCNA), we constructed co-expression networks and modules using the dysregulated genes related to H2AZ1 overexpression. To construct a scale-free network, we set the soft threshold power β to 8 (R2=0.85), and DEGs with similar expression patterns were clustered into 16 co-expression modules (Figure 2E, 2F). Pearson’s correlation coefficients were used to analyze the interactions among these co-expression modules, and the branches of the dendrogram were grouped according to the relatedness of the eigengenes (Figure 2G). We further analyzed the correlation between each module, H2AZ1, and clinical features (Figure 2H) and found that the black module had the most significant correlation with H2AZ1 (R=0.82, p=6e-95), while it was negatively correlated with overall survival (OS) (R=-0.12, p=3e-05). Therefore, co-expression in the black modules might be a key factor in the poor prognosis of H2AZ1-mediated HCC.

### Biological functions and signaling pathways associated with H2AZ1 dysregulation in HCC

**Figure 3:**
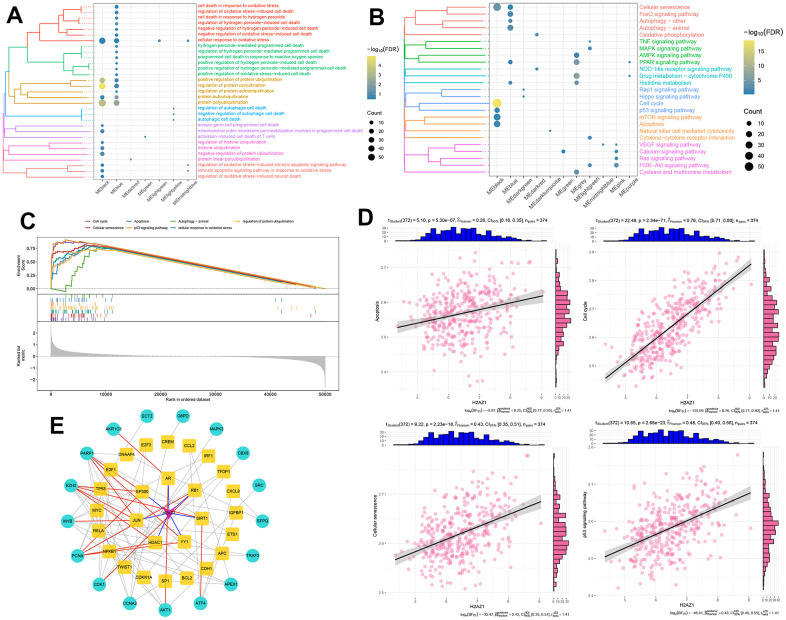
**Biological functions and signaling pathways involved in the significant regulatory modules of H2AZ1.** (**A**) Clustered bubble plot showing biological functions significantly regulated by H2AZ1. (**B**) Clustered bubble plot showing signaling pathways significantly regulated by H2AZ1. (**C**) Gene set enrichment analysis map showing signaling pathways significantly activated by H2AZ1. (**D**) Correlation between H2AZ1 expression and apoptosis, cell cycle, cell senescence, and the p53 signaling pathway gene set. (**E**) Network of H2AZ1 regulating cellular response to oxidative stress through transcription factors.

Subsequently, we performed a functional enrichment analysis of the module genes. The results showed that the black module genes were significantly enriched in biological processes related to oxidative phosphorylation and ubiquitination (P<0.05, Figure 3A), as well as pathways such as cellular senescence, cell cycle, P53 signaling pathway, and apoptosis (P<0.05, Figure 3B). Using gene set enrichment analysis (GSEA), we verified the significant activation of gene sets linked to cellular senescence, cell cycle, P53 signaling pathway, and apoptosis in the H2AZ1 high expression group (Figure 3C), suggesting that these pathways may be closely associated with H2AZ1-mediated HCC development. Correlation analysis showed that H2AZ1 expression in HCC was significantly and positively correlated with the gene set scores for apoptosis, cell cycle, cellular senescence, and P53 signaling pathway (Figure 3D). In addition, using pivot analysis, we explored the regulation of H2AZ1 in cellular responses to oxidative stress in black module genes (Supplementary Table 1) and constructed a protein-protein interaction network. These results indicated that H2AZ1 may regulate cellular responses to oxidative stress gene sets by regulating transcription factors such as JUN, YY1, and SIRT1 (Figure 3E). H2AZ1 overexpression in HCC may, thus, affect the development of tumor cells by regulating cell apoptosis, cell cycle, and aging.

### H2AZ1-based index model shows significant prognostic potential in HCC

**Figure 4:**
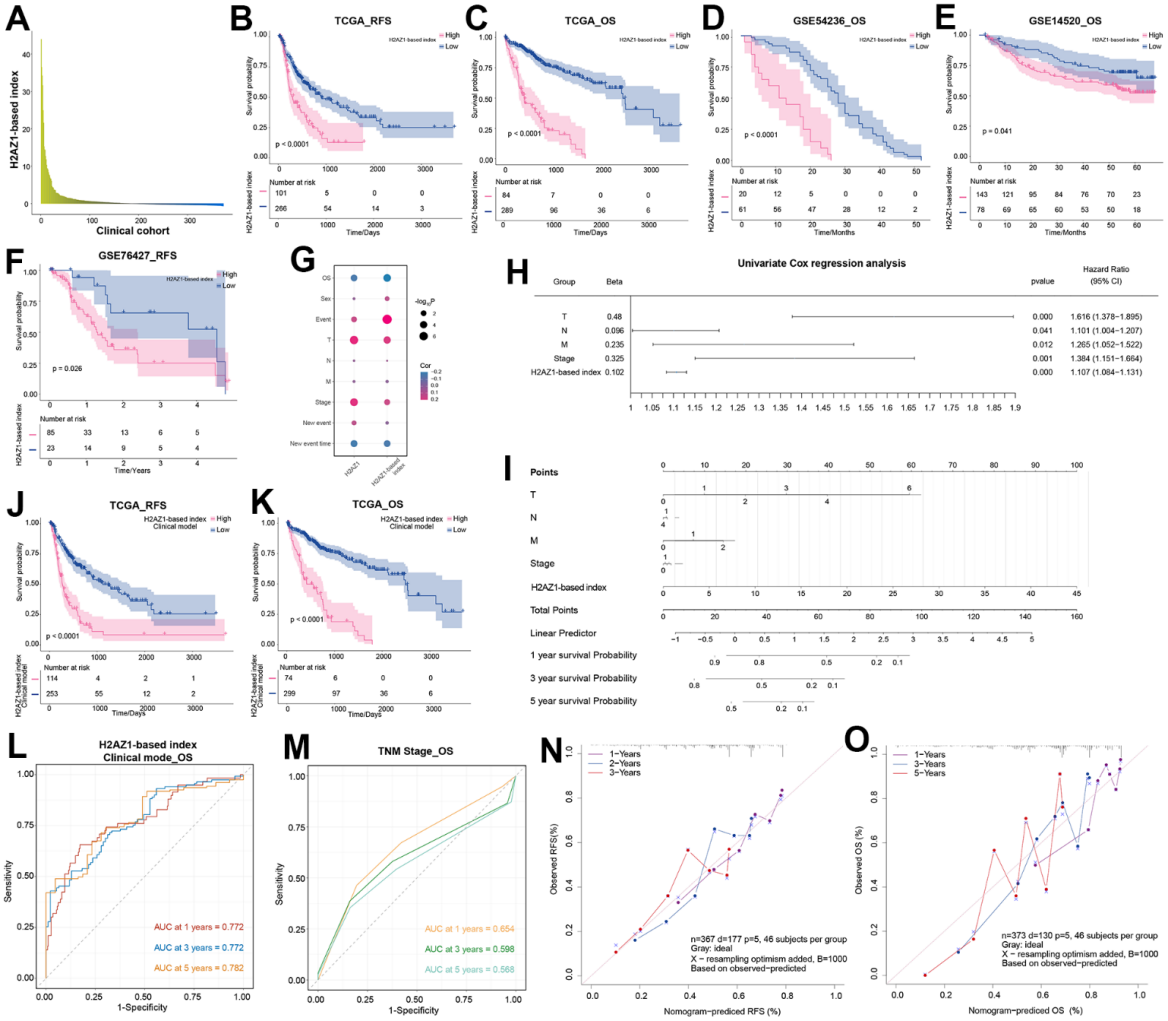
**Exploring the prognostic efficacy of H2AZ1-based index clinical model in hepatocellular carcinoma.** (**A**) Bar graph showing the expression of H2AZ1-based index in the clinical cohort of patients with hepatocellular carcinoma. (**B**) Recurrence-free survival (RFS) survival curve of the H2AZ1-based index in TCGA-LIHC data. (**C**) Overall survival (OS) survival curve of the H2AZ1-based index in TCGA-LIHC data. (**D**) Survival curves demonstrate the OS prognostic potential of the H2AZ1-based index in the GSE54236 data set. (**E**) Survival curves demonstrate the OS prognostic potential of the H2AZ1-based index in the GSE14520 data set. (**F**) Survival curves demonstrate the RFS prognostic potential of the H2AZ1-based index in the GSE76427 data set. (**G**) Bubble plot showing the correlation between the H2AZ1-based index and clinical indicators. (**H**) Forest plot showing the univariate prognostic power of the H2AZ1-based index and clinical indicators. (**I**) Nomogram showing H2AZ1-based index clinical model. (**J**) Survival curves demonstrating RFS prognostic potential of the H2AZ1-based index clinical model. (**K**) Survival curves demonstrate the OS prognostic potential of the H2AZ1-based index clinical model. (**L**) Time-dependent receiver operator characteristic (ROC) curve of the H2AZ1-based index clinical model. (**M**) Time-dependent ROC curve of TNM stage. (**N**) Calibration curve demonstrating RFS prognostic potential of H2AZ1-based index clinical model. (**O**) Calibration curve demonstrating the OS prognostic potential of the H2AZ1-based index clinical model.

To explore the potential clinical roles of H2AZ1 and its related dysregulated genes in HCC, we performed survival analysis on these genes and selected the top 50 genes most significantly associated with HCC overall survival (OS) and recurrence-free survival (RFS) (Supplementary Table 2). We further screened these genes by univariate analysis, excluding KAT2A (P=0.029) based on a significance threshold of P<0.01, and constructed the H2AZ1-based index by Cox multivariate analysis (Supplementary Table 3). We explored the expression of this index in a clinical cohort of patients with HCC (Figure 4A). Moreover, survival curve analysis revealed that a high H2AZ1-based index was associated with poor OS and RFS in HCC (Figure 4B, 4C), which was verified in HCC-independent cohorts, such as GSE54236, GSE14520, and GSE76427 (Figure 4D–4F). Notably, this index showed a significant negative correlation with the OS of patients with HCC (Figure 4G). Univariate analysis showed that H2AZ1-based indices, such as tumor size (T), lymph node involvement (N), distant metastasis (M), and stage, could serve as independent risk factors for HCC (Figure 4H). Subsequently, based on clinical characteristics such as TNM staging of patients with HCC and the H2AZ1 index, we further established a clinical model and represented it using a nomogram (Figure 4I). Survival analysis showed that this clinical model had predictive potential for OS and RFS in HCC (Figure 4J, 4K), and the receiver operating characteristic (ROC) curve showed that this clinical model had higher accuracy than the TNM model (Figure 4L, 4M). Furthermore, the calibration curve validated the predictive accuracy of the nomogram (Figures 4N, 4O).

### Poor prognosis in the high H2AZ1-based index group may be associated with the stemness of the tumor

**Figure 5:**
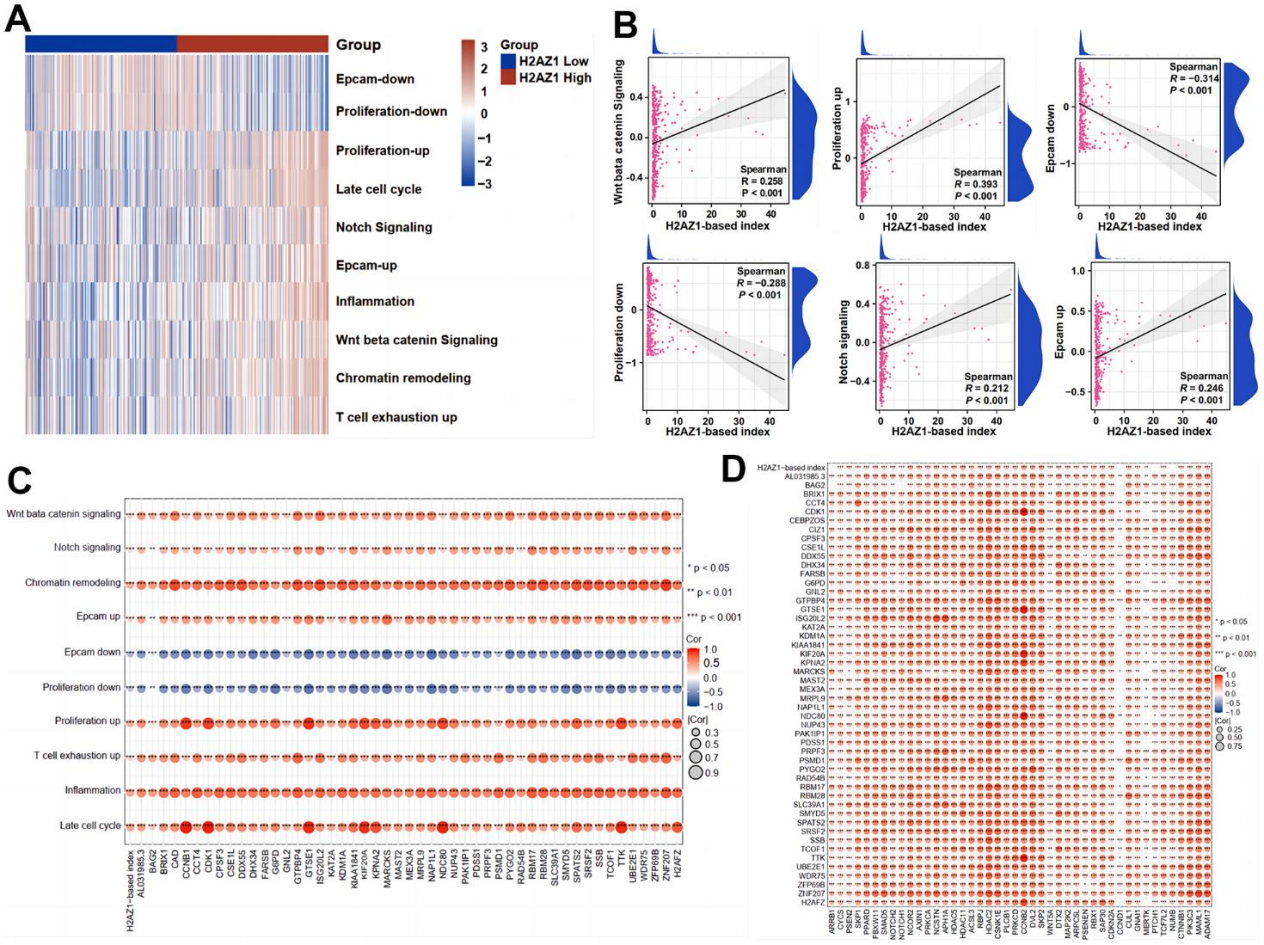
**Expression of stemness-related pathway scores in high and low H2AZ1-based index groups.** (**A**) Heat map showing the expression of stemness-related pathway scores in high and low H2AZ1-based index groups. (**B**) Scatter plot showing the correlation between H2AZ1-based index and tumor stemness-related pathway score. (**C**) Bubble chart showing the correlation between H2AZ1-based index genes and tumor stemness-related pathway scores. (**D**) Bubble chart showing the correlation between H2AZ1-based index genes and tumor stemness-related genes.

To explore the relationship between H2AZ1-based index genes and tumor stemness, we used the gene set variation analysis (GSVA) algorithm to assess stemness-related gene sets (Supplementary Table 4). The analysis showed that the high H2AZ1-based index group had higher scores in stemness, particularly in the Wnt and Notch signaling pathways (Figure 5A). In addition, correlation analysis showed that the high H2AZ1-based index group was positively correlated with the Wnt signaling pathway, Notch signaling pathway, Epcam upregulation, and proliferation upregulation signals. Conversely, it was negatively correlated with Epcam downregulation and proliferation downregulation signals. These findings suggest that the poor prognosis observed in the high H2AZ1-based index group might be related to the activation of tumor stemness-related pathways (Figure 5B). Further correlation analysis indicated that the genes of the H2AZ1-based index were positively correlated with tumor stemness pathways and stemness-related genes (Figure 5C, 5D). These results suggest that the increased expression of H2AZ1 index genes in HCC may enhance the stemness of tumor cells, thereby promoting HCC progression.

### Identification of upstream regulators of H2AZ1-based index gene sets

**Figure 6:**
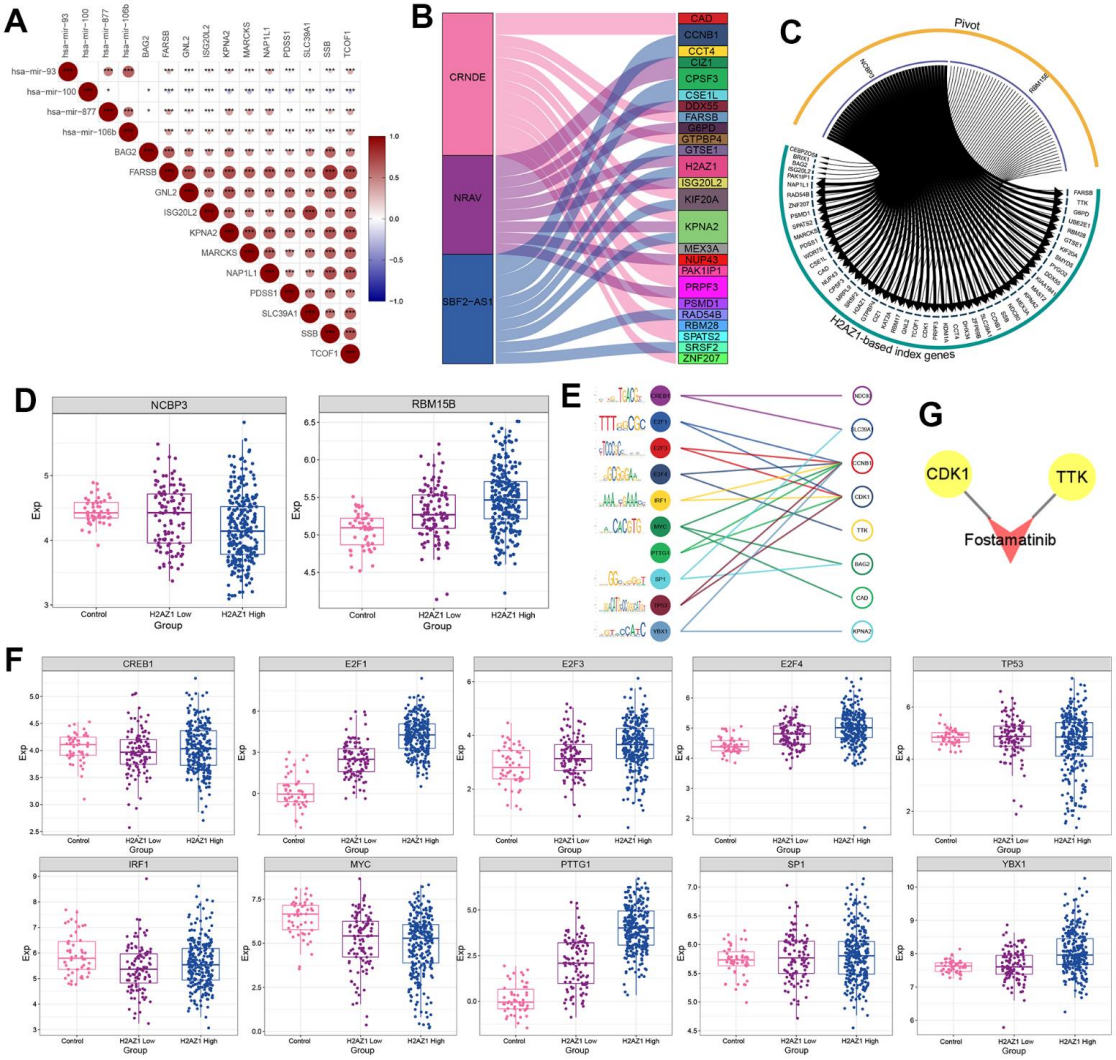
**Transcription factor prediction based on H2AZ1-based index.** (**A**) Triangular correlation heatmap - miRNAs showing the regulatory role of miRNAs on the H2AZ1-based index gene set. (**B**) Sankey diagram showing the regulation of lncRNAs on the H2AZ1-based index gene set. (**C**) Circle network diagram showing the regulation of RNA-binding proteins (RBPs) on the H2AZ1-based index gene set. (**D**) Box plots showing the transcriptional expression levels of some RBPs. (**E**) Bubble line-motif logo showing the regulatory effect of TF on the H2AZ1-based index gene set. (**F**) Box plots showing transcriptional expression levels of transcription factors (TFs). (**G**) Network diagram showing potential drug targets of the H2AZ1-based index gene set.

Subsequently, we explored the upstream regulators of the H2AZ1-based index gene set, including miRNAs, lncRNAs, RNA-binding proteins (RBPs), and transcription factors (TFs), using pivot analysis. The results showed that the upstream miRNAs regulating the H2AZ1-based index gene set included hsa-mir-93, hsa-mir-100, and hsa-mir- 877 (Figure 6A). The upstream lncRNAs included CRND1, NRAV, and SBF2-AS1 (Figure 6B), and the RBPs included NCBP3 and RBM15B (Figure 6C). Notably, NCBP3 targeted UBE2E1, a gene encoding the ubiquitin-conjugating enzyme E2E1. The expression level of NCBP3 was low in the H2AZ1 high-expression group, whereas the expression level of RBM15B was increased in the control, H2AZ1 low-expression, and H2AZ1 high-expression groups (Figure 6D). In addition, 10 TFs, including CREB1, E2F1, and E2F3 regulated genes in the H2AZ1-based index (Figure 6E, 6F). We also explored potential drug targets in the H2AZ1-based index gene set, including CDK1 and TTK (Figure 6G).

### Multi-omics global regulatory network of H2AZ1-based index genes

**Figure 7:**
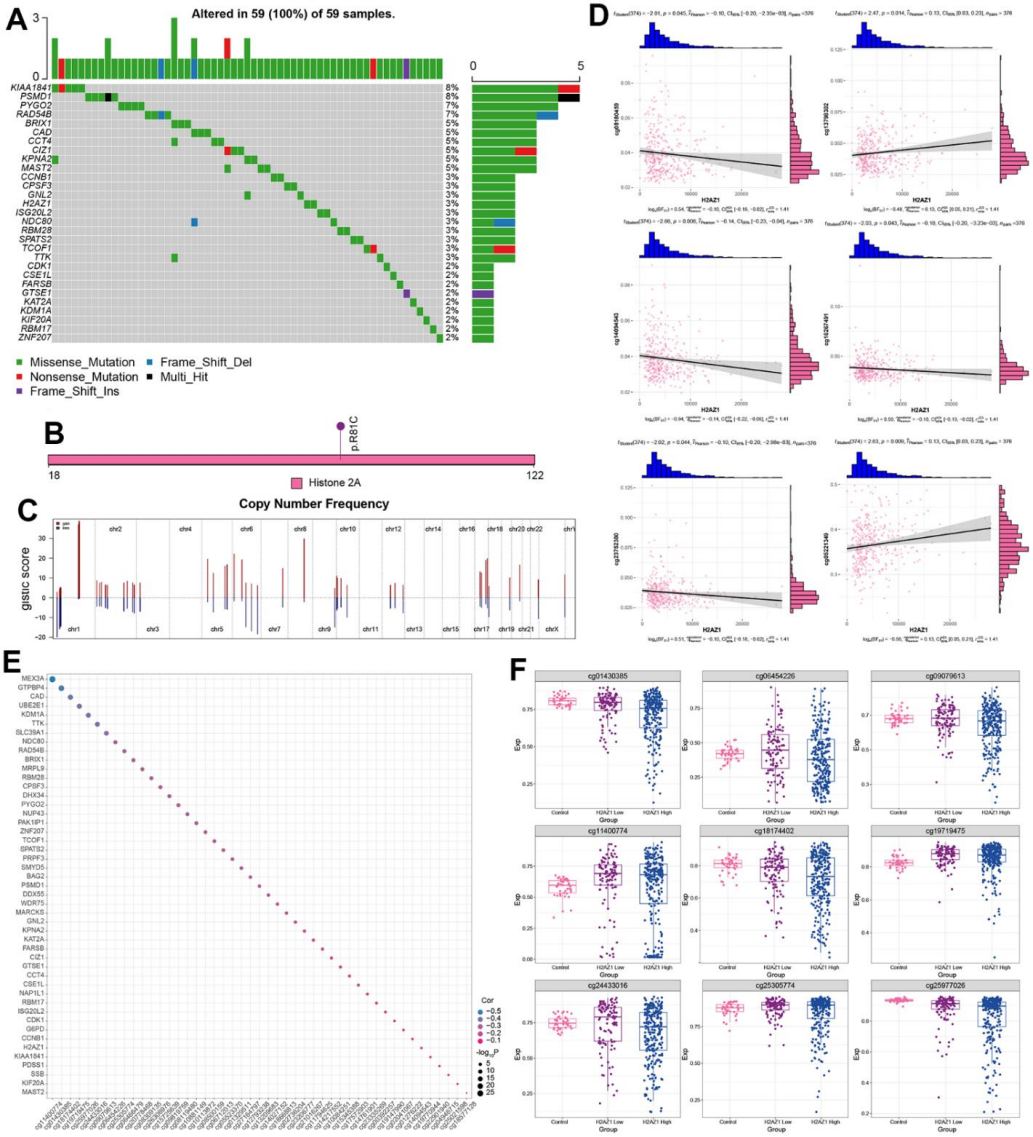
**Multi-omics landscape of the global regulatory network of H2AZ1-based index genes.** (**A**) Waterfall plot showing the mutational landscape (SNP) of H2AZ1-based index gene global regulatory network in liver cancer. (**B**) Lollipop diagram showing details of H2AZ1 mutation in liver cancer. (**C**) Chromosome bar graph showing the copy number spectrum of H2AZ1-based index gene global regulatory network in liver cancer. (**D**) Scatter plots of serial correlations showing the correlation of H2AZ1-based index gene global regulatory network in the methylation modification level and transcription level of liver cancer. (**E**) Bubble plot showing H2AZ1-based index genes are regulated by methylation. (**F**) Series of box plots showing the methylation level of H2AZ1-based index gene global regulatory network in liver cancer.

Additionally, we explored somatic mutations in H2AZ1-based index genes in HCC. Mutations in KIAA1841, PSMD1, PYGO2, and RAD54B were the most frequent, and H2AZ1 mutations occurred at a frequency of 3% (Figure 7A). The mutation site of H2AZ1 is shown in Figure 7B. In the H2AZ1-based index gene global regulatory network, an increase in copy number and deletions was observed (Figure 7C). Correlation analysis between the methylation modification level and the H2AZ1-based index gene showed that the expression of H2AZ1 was negatively correlated with the methylation sites cg08180459, cg14094543, cg16267491, and cg23752380 (Figure 7D). Some genes, such as MEX3A, were significantly negatively correlated with the methylation sites (Figure 7E). Finally, the expression profiles of some methylated sites that were significantly differentially expressed in the control and H2AZ1 high/low expression groups are shown in Figure 7F.

### Cellular experimental validation of the potential function of H2AZ1 in HCC

**Figure 8:**
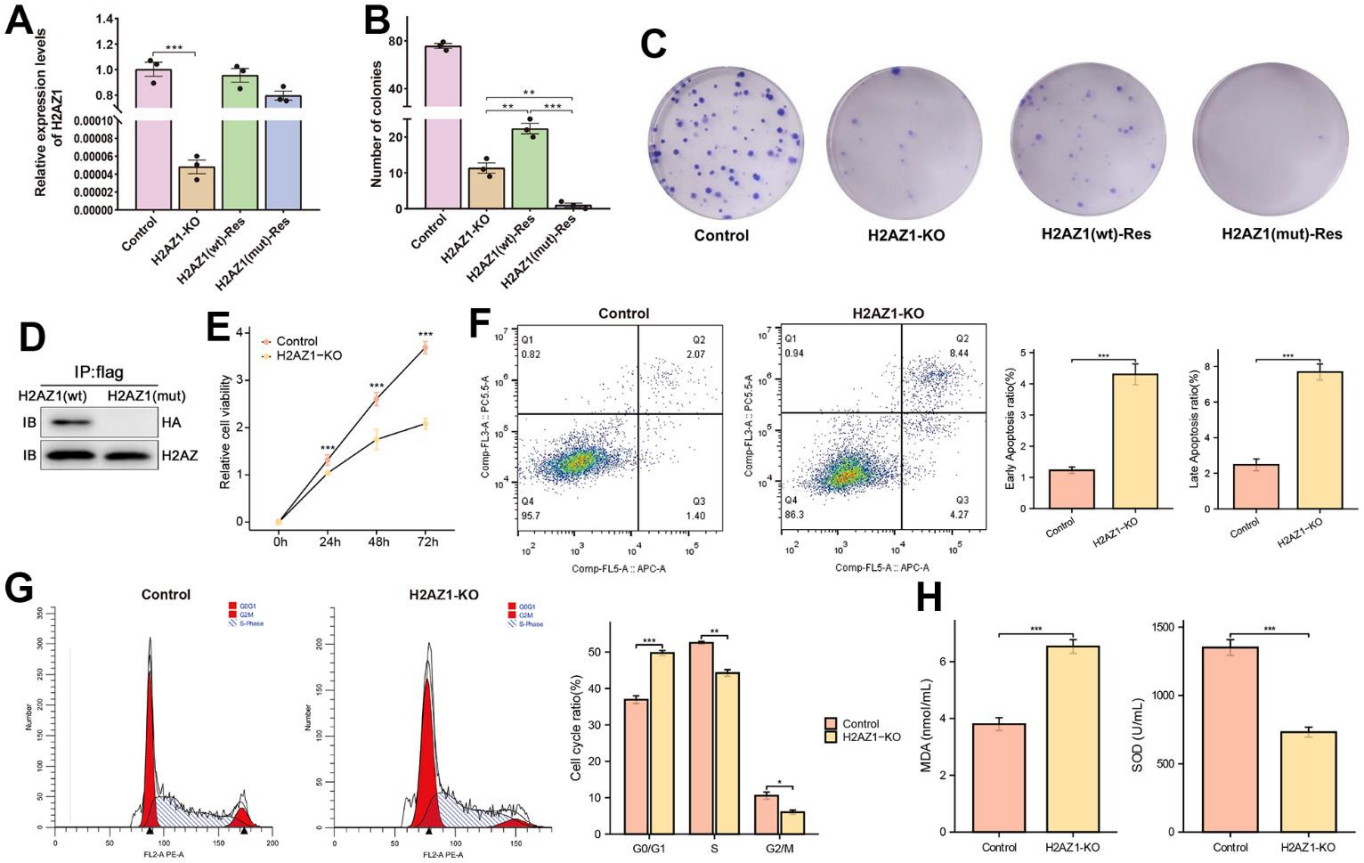
**Cell models of H2AZ1 ubiquitination variants of Flag-H2AZ1 (WT) and Flag-H2AZ1 (MUT).** (**A**) Expression of H2AZ1 in the control, H2AZ1-KO, and rescued cell models. (**B**) Calculation of colony numbers in the control and rescued cell models. Colony numbers comprising more than 50 cells were calculated. (**C**) Representative images of colony formation assay. (**D**) Western blotting indicates impaired ubiquitination function after site mutation in H2AZ1. (**E**) Cellular activity of Control, H2AZ1-KO. (**F**) Apoptosis ratio of Control, H2AZ1-KO. (**G**) Cell cycle of Control, H2AZ1-KO. (**H**) MDA level and SOD activity of Control, H2AZ1-KO.

The above findings showed that dysregulated genes associated with H2AZ1 were significantly enriched in biological processes and pathways such as ubiquitination, oxidative stress, apoptosis, and the cell cycle, suggesting that H2AZ1 may influence HCC development through these pathways. Previous studies have demonstrated the ubiquitination properties of H2AZ. Therefore, we hypothesized a potential close association between H2AZ and ubiquitination in HCC. Therefore, we constructed an *in vitro* cell model by introducing point mutations at the H2AZ1 ubiquitination sites (K120, K121, and K125) [25–27] using homologous recombination with CRISPR/Cas9 technology. However, following numerous attempts, we could not obtain single clones with these point mutations, suggesting that ubiquitination-deficient cells might be too weak to survive. Therefore, an alternative approach was employed using wild-type and ubiquitination-deficient mutant H2AZ1 overexpression constructs to establish the corresponding cell models. The expression levels of H2AZ1 in these constructed cell models were confirmed using quantitative real-time PCR (Figure 8A). We observed a significant decrease in the colony-forming ability of cancer cells after H2AZ1 knockout (Figure 8B). Notably, when the H2AZ1-KO cell model was supplemented with H2AZ1(wt) and H2AZ1(mut) constructs, overexpression of H2AZ1(wt) partially restored the colony-forming ability, whereas overexpression of H2AZ1(mut) failed to do so (Figure 8C). Additionally, immunoprecipitation (IP) analyses confirmed successful precipitation of ubiquitinated HA plasmids from H2AZ (wt) cells, displaying ubiquitination characteristics, whereas mutant H2AZ (mut) cells could not achieve this (Figure 8D).

Subsequently, we conducted CCK8 proliferation, cell cycle, and apoptosis assays to verify the biological effects of H2AZ1 on HCC cells. The cellular experiments showed that H2AZ1 knockdown increased apoptosis ratio and altered cell cycle transitions in HCC cells (Figure 8E–8G). Moreover, H2AZ1 knockdown resulted in a decrease in superoxide dismutase (SOD) activity and an increase in malondialdehyde (MDA) levels, suggesting that changes in H2AZ1 expression could affect oxidative stress in tumor cells (Figure 8H).

## DISCUSSION

As HCC is a malignancy with an extremely high mortality rate when diagnosed in late-stage cases [28], exploring HCC-related genes can help identify new and promising prognostic biomarkers and drug targets to improve the clinical prognosis of patients with hepatocellular carcinoma. In this study, we found that dysregulated HCC genes associated with H2AZ1 overexpression were significantly enriched in key biological processes and pathways, such as ubiquitination, oxidative stress, apoptosis, and cell cycle. Our findings indicate that ubiquitinated H2AZ1 may partially restore the colony-forming capacity of HCC cells. Furthermore, we observed that H2AZ1 knockdown significantly influenced tumor cell proliferation, cell cycle transition, and apoptosis, which may provide a foundation for understanding the biological functions of H2AZ1 in HCC.

Oxidative stress contributes to the development of many diseases and is an important factor in carcinogenesis [29, 30]. Notably, oxidative stress is a key factor in the initiation and progression of HCC under various pathological conditions [31]. In this study, we found that H2AZ1 indirectly regulates oxidative stress by regulating the expression of YY1, JUN, and SIRT1. SIRT1 overexpression can reduce oxidative stress [32], and YY1 plays an important role in preventing pathological oxidative stress [33]. This suggests that the overexpression of H2AZ1 in hepatocellular carcinoma may indirectly regulate oxidative stress by targeting TFs such as SIRT1 and YY1. Cell experiments showed that H2AZ1 knockdown led to a decrease in SOD activity and an increase in MDA level in HCC cells, providing preliminary evidence that H2AZ1 overexpression promotes oxidative stress in HCC cells. However, the specific targets of H2AZ1 require further study.

Ubiquitination is an important post-translational modification involved in regulating inflammatory cell death and is closely associated with cancer development [34, 35]. It has emerged as a key mediator and regulator of signaling in cell death and inflammation [36]. To investigate the potential role of H2AZ1 ubiquitination, we constructed an *in vitro* cell model with point mutations at the H2AZ1 ubiquitination sites using homologous recombination with CRISPR/Cas9 technology. However, we were unable to obtain a clone with the point mutations. Histone variants are important regulators of embryonic development because their knockdown is often lethal. Successfully achieving knockdown in cultured cells can be challenging [13]. Therefore, this suggests that ubiquitination of H2AZ1 is also critical for its normal regulatory function in HCC cell activities. Using alternative cellular models, we observed a significant decrease in cancer cell colony-forming ability after H2AZ1 knockout, and overexpression of H2AZ1(WT) partially restored the colony-forming ability, whereas overexpression of H2AZ1(MUT) did not. Additionally, H2AZ1 knockdown reduced HCC cell viability, increased apoptosis, and caused cell cycle arrest at the G0/G1 phase. H2AZ1 regulates the expression of cell cycle genes such as Myc and Ki-67, and its depletion leads to G1 arrest and cellular senescence, consistent with our main findings [37]. These studies indicate that H2AZ1 may regulate the HCC cell cycle and inhibit apoptosis.

Through correlation analysis, we found that high expression of the H2AZ1 index gene in HCC may enhance the stemness of tumor cells and promote the progression of HCC. In addition, we explored the upstream regulators of the H2AZ1-based index gene set. Some lncRNAs, including NRAV and SBF2-AS1, have been associated with predicting immune checkpoint blockade and HCC prognosis [38, 39]. High expression levels of RBPs, such as RBM15B, have also been associated with poor prognosis in patients with HCC and the promotion of cancer cell proliferation and invasion [40]. Furthermore, we identified two targets of fostamatinib, CDK1 and TTK, in the H2AZ1-based index gene set. Dysregulation of the cyclin-dependent kinase CDK1 has been closely linked to tumorigenesis, and its activation plays a key role in various cancers [41]; TTK is a dual-specificity protein kinase involved in cell proliferation and division [42] and is critical for chromosome arrangement at centromeres during mitosis and centrosome duplication [43]. CDK1 is overexpressed in HCC and associated with poor OS [44]. Moreover, TTK inhibitors induce aneuploidy and senescence in HCC cells [45], effectively eliminating tumors. Fostamatinib is an antitumor drug with HCC-related targets [46]. This evidence indicates that CDK1 and TTK are important targets in HCC cells, suggesting that they may be potential therapeutic drugs for HCC.

Nonetheless, it is important to acknowledge the limitations of this study. The sample size in our analysis was relatively small, and further sample expansion is needed to verify these results. Additionally, the dysregulated genes related to H2AZ1 were primarily identified through bioinformatics analysis. The H2AZ1-based index identified the top 50 genes showing the most significant correlation with HCC prognosis in the module of interest. Thus, the inclusion or exclusion of additional genes could lead to variations in this index. Further molecular experiments are required to validate the specific mechanisms of action of H2AZ1 and related dysregulated genes in HCC.

In conclusion, we have elucidated the potential roles of H2AZ1 in HCC and constructed an H2AZ1-based index capable of predicting the survival of patients with HCC. Therefore, future research should focus on identifying and investigating the specific targets of H2AZ1 for tumor cell inhibition by knockdown or overexpression.

## MATERIALS AND METHODS

### Data sources

The Cancer Genome Atlas-liver hepatocellular carcinoma (TCGA-LIHC) dataset, consisting of 374 HCC samples and 50 paracancerous control samples, was obtained from TCGA database (https://www.cancer.gov/) [47]. The data were normalized using the limma package [48]. Principal component analysis (PCA) was performed to differentiate HCC samples from normal samples based on their gene expression patterns [49]. In addition, we obtained HCC-related transcriptome data, including GSE54236, GSE14520, and GSE76427, from the Gene Expression Omnibus (GEO) database to validate the prognostic potential of the H2AZ1-based index in HCC. All clinical data and mRNA expression data were retrieved and downloaded from the GEO database and TCGA, and the patients involved in the databases have provided their consent.

### Differential gene expression analysis of genes and miRNAs

To explore the dysregulated genes affected by high H2AZ1 expression in HCC, differential expression analysis of miRNAs and mRNA was conducted using the limma package [48]. This analysis specifically focused on HCC control samples and samples with high and low expression levels of H2AZ1. DEGs that consistently exhibited upregulation or downregulation in both groups were identified as deregulated genes influenced by high H2AZ1 expression in HCC.

### Weighted correlation network analysis

Weighted gene co-expression network analysis (WGCNA) is widely used to construct scale-free networks using gene expression data. To examine the effect of high H2AZ1 expression on gene deregulation in HCC, we used the WGCNA package [50]. The analysis used deregulated genes affected by high H2AZ1 expression in HCC. The hclust function of the WGCNA package was used to cluster samples. Subsequently, a suitable soft-thresholding power was determined to generate a proximity matrix that best matched the scale-free network characteristics of gene distribution (R2 = 0.85). Moreover, a heatmap illustrating the correlations between modules and phenotypes was generated to identify significant module-phenotype correlations. In this study, the modules that exhibited the most significant positive correlation with HCC were designated as candidate modules.

### Functional enrichment analysis of co-expression modules

The clusterProfiler package [51] was used to perform Gene Ontology (GO) and Kyoto Encyclopedia of Genes and Genomes (KEGG) pathway enrichment analyses of modular genes to explore their potential biological functions. The reference gene set used for this analysis was c5.bp.v7.0.entrez.gmt and c2.cp.kegg.v7.0.symbols.gmt from the Molecular Signature Database (MSigDB) [52]. Subsequently, the phenotype and full gene expression profiles were input into the Gene Set Enrichment Analysis (GSEA) [53] to verify the signaling pathways that were significantly activated or inhibited by these genes.

In addition, we obtained gene sets of interest from the KEGG and GO databases and performed a single-sample gene set enrichment analysis (ssGSEA) using the GSVA package [54]. The correlation between H2AZ1 and gene set scores was subsequently calculated by correlation analysis, and a P-value < 0.05 was considered statistically significant.

### Construction and validation of H2AZ1-based index clinical model

The modules that were the most significantly correlated with H2AZ1 were identified using WGNCA. Kaplan–Meier (KM) survival analysis was performed on these module genes to identify genes associated with OS and RFS in HCC. Seven samples were excluded from the RFS analysis and one sample from the OS analysis due to missing survival data in TCGA-LIHC dataset. Univariate and multivariate Cox regression analyses were performed on the 50 most significant genes and H2AZ1 expression to establish the H2AZ1-based index. A clinical model based on the H2AZ1 index was further refined using alignment diagrams. The sensitivity and specificity of this model were evaluated using time-dependent ROC analysis with the timeROC package. Additionally, the predictive ability of the model for patients with HCC prognosis was assessed through nomograms and calibration curves, implemented with the “rms” package.

### Upstream regulators and potential drug targets of H2AZ1-based index genes

H2AZ1-based index genes were subjected to pivot analysis to determine the upstream miRNAs, lncRNAs, RNA binding proteins (RBPs), and transcription factors (TFs) that regulate these genes. In addition, this analysis aimed to investigate potential drug targets. Pivot analysis utilized data from the RNAInter, TRRUST, STRING, and DrugBank databases [55–58] to identify regulators that interact with the target genes through hypergeometric tests. Statistical significance was set at P <0.05.

### Construction of a multi-omics regulatory landscape of H2AZ1-based index genes

To investigate the single nucleotide polymorphisms (SNPs) of H2AZ1-based index genes in HCC, we utilized TCGA-LIHC data and employed the R package maftools [59] to visualize the SNPs of these genes and the mutation details of H2AZ1. Subsequently, we determined the copy number spectra of these genes in patients with HCC. In addition, correlation analysis was used to explore the correlation between methylation modifications and the transcription levels of H2AZ1-based index genes.

### Cell culture, rescue experiment, and colony formation assay

HepG2 cells were purchased from the Cell Bank of the Chinese Academy of Sciences (Shanghai, China) and cultured in Dulbecco’s modified Eagle’s medium (DMEM, Gibco, China) supplemented with 10% fetal bovine serum (FBS, Gibco, Australia) and 1% streptomycin-penicillin. The cells were maintained at 37° C in a humidified atmosphere containing 5% CO_2_. A HepG2 H2AZ1-KO cell model was successfully established as previously described [23]. Subsequent rescue experiments were performed in HepG2 H2AZ1-KO cells by overexpressing H2AZ1 (WT) with ubiquitination and H2AZ1 (MUT) without ubiquitination. The expression of H2AZ was assessed by PCR in different cell models. Primer sequences were as follows: 5’-GCAGTTTGAATCGCGGTG-3’ (forward) and 5’-GAGTCCTTTCCAGCCTTACC-3’ (reverse) for H2AZ1; 5’-CTCCATCCTGGCCTCGCTGT-3’ (forward) and 5’-GCTGTCACCTTCACCGTTCC-3’ (forward) for Actin. Colony formation assays were performed to assess the effect of H2AZ1 ubiquitination on the ability of cells to form colonies. In six well plates, approximately 500 cells per well were seeded for the HepG2 H2AZ1-KO, H2AZ1 (WT), and H2AZ1 (MUT) cell lines, followed by incubation at 37° C in 5% CO_2_. After 2 weeks, the cells were washed with PBS, fixed with 4% paraformaldehyde, and stained with a 0.1% Giemsa dye solution at room temperature. The colonies were observed under a microscope, and a colony comprising more than 50 cells was considered as one positive colony and captured as a photograph. Three replicates were prepared for each group, and the experiment was repeated thrice. The collected data were statistically analyzed using GraphPad software (GraphPad Prism 8).

### Immunoprecipitation (IP) and immunoblot analysis

The HA-ubiquitin plasmid was co-transfected with Flag-H2AZ1(WT) and Flag-H2AZ1(MUT) in HEK293T cells respectively. Then, the histones were extracted, and IP was performed using IP buffer (10mM Tris-HCl PH 7.4, 10mM NaCl, 0.2 mM EDTA) followed by incubation with Monoclonal anti-Flag®M2-conjugated agarose beads at 4° C overnight. The immunoprecipitated complexes were washed and precipitated thrice with IP buffer. Flag-H2AZ1(WT) and Flag-H2AZ1(MUT) were pulled down and analyzed by immunoblotting. HA-tag and H2AZ antibodies were used to detect the IP products. The antibodies used were anti-FLAG ®M2 (F3165, Sigma, USA) and anti-H2A, Z antibody (ab4174, Abcam, UK), and anti-HA tag antibody (ab9110, Abcam, UK). Three independent experiments were conducted, and triplicate samples from each group were analyzed. The density of the immunoblot bands was quantified using ImageJ software (ImageJ 1.8.0, National Institutes of Health, USA) for data analysis.

### CCK-8 cell proliferation and activity detection

The Cell Counting Kit-8 (CCK8) method was used to measure cell viability. Briefly, 100 μL of cell suspension cells were inoculated on 96-well plates. Cell viability was assessed using the CCK8 reagent (MCE) according to the manufacturer’s protocols. The absorbance at 450 nm was recorded using a microplate reader. Three replicates were prepared for each group, and the experiment was repeated three times. The collected data were statistically analyzed using the SPSS software (SPSS 25).

### Cell apoptosis and cell cycle

Apoptosis was induced according to the experimental protocol, and 1–10 × 10^5^ cells were collected, stained with Annexin V-APC and 7-AAD, and loaded to the machine for flow analysis. In addition, cell cycle distribution was measured using the Cell Cycle Analysis Kit. Cells (1 × 10^5^ cells/mL, 2 mL) were seeded in six-well plates. After treatment, the cells were fixed overnight in 70% ice-cold ethanol at 4° C. Subsequently, the cells were incubated with propidium working solution containing 10 μL RNase A for 30 min at 37° C. Red fluorescence was detected using a flow cytometer (BD FACSCantoTM II) at an excitation wavelength of 488 nm, detecting light scattering. Three independent experiments were performed, and triplicate samples were analyzed for each group. Cell cycle data were analyzed using the MODFIT software (ModFit LT 5.0), whereas apoptosis data were analyzed using the FLOWJO software (FlowJo V10).

### Enzyme-linked immunosorbent assay (ELISA) detection of malondialdehyde (MDA) and superoxide dismutase (SOD)

To detect MDA level and SOD activity in H2AZ1 knockdown and control cells, MDA (MLbio-ml950271) and SOD (JYM2065Hu) were purchased from MLBio (USA) and Wuhan Colorful Gene Biological Technology Co., Ltd., (China) respectively. Standard and sample wells were prepared as instructed, and different concentrations of standards and samples were added to each well. After the reaction, the optical density (OD) at 450 nm was measured, and a standard curve was drawn using the standard product. MDA level and SOD activity of each sample were calculated using a curve equation. Three replicate wells were used for each group, and the experiment was repeated three times. Data were analyzed using the SPSS software (version 25).

### Data analysis and statistics

The bioinformatics analysis carried out in this study, such as WGCNA, KM survival, and ROC curve analysis was performed using the BioinforCloud platform (http://www.bioinforcloud.org.cn), Xiantao website (https://www.xiantaozi.com/) and RStudio software. An area under the curve (AUC) >0.7 indicated that the model has good diagnostic potential. The normality and homogeneity of variance of the data distribution were compared in groups using the T-test, one-way ANOVA, or Wilcoxon rank sum test, respectively. Variables that satisfy the normal distribution are expressed as the mean ± standard deviation, and variables that do not satisfy the normal distribution are expressed as the median (interquartile). * P < 0.05, ** P < 0.01, *** P < 0.001, ns indicates no significant difference. P < 0.05 was considered statistically significant.

### Data availability

The data used to support the findings of this study are included within the article, and are available from the corresponding author upon request.

## Supplementary Material

Supplementary Tables 1-3

Supplementary Table 4
