# Supplementary material for: Identification and validation of an H2AZ1-based index model: a novel prognostic tool for hepatocellular carcinoma
Source: Aging (Albany NY). 2024 Feb 1;16(3):2542–62. doi: 10.18632/aging.205497 (PMC10911386; doi:10.18632/aging.205497)
Supplement: Supplementary Tables 1-3 [file aging-16-205497-s001.pdf]

SUPPLEMENTARY TABLES

Supplementary Table 1. Cellular response to oxidative stress gene set enriched by black module genes.

Gene
AIFM2
AKR1C3
AKT1
APEX1
ATF4
ATG7
CBX8
CCNA2
CDK1
ECT2
EZH2
FANCD2
G6PD
GPX1
MAPK3
MELK
MPV17
MYB
NME2
NONO
NUDT2
P4HB
PARP1
PCNA
PLA2R1
PRDX1
PRDX5
PRKCD
PSAP
PYCR1
PYCR2
RACK1
ROMO1
RPS3
SFPQ
SRC
STK25
STX4
TLDC2
TRAF2
ZNF580

Supplementary Table 2. H2AZ1-associated HCC prognostic index gene set.

Gene
AL031985.3
BAG2
BRIX1
CAD
CCNB1
CCT4
CDK1
CEBPZOS
CIZ1
CPSF3
CSE1L
DDX55
DHX34
FARSB
G6PD
GNL2
GTPBP4
GTSE1
ISG20L2
KDM1A
KIAA1841
KIF20A
KPNA2
MARCKS
MAST2
MEX3A
MRPL9
NAP1L1
NDC80
NUP43
PAK1IP1
PDSS1
PRPF3
PSMD1
PYGO2
RAD54B
RBM17
RBM28
SLC39A1
SMYD5
SPATS2
SRSF2
SSB
TCOF1
TTK

UBE2E1
WDR75
ZFP69B
ZNF207
H2AFZ

Supplementary Table 3. H2AZ1-based index model equation.

H2AZ1-based index =
(0.00207)*AL031985.3+(0.00033)*BAG2+(0.00009)*BRIX1+(0.00015)*CAD+(-0.00014)*CCNB1+(0)*CCT4+(-0.00026)*CDK1+(0.00056)*CEBPZOS+(-0.00022)*CIZ1+(-0.00017)*CPSF3+(0.00004)*CSE1L+(0.00065)*DDX55+(-0.00006)*DHX34+(0.00003)*FARSB+(0.00006)*G6PD+(-0.00002)*GNL2+(-0.00004)*GTPBP4+(0.00024)*GTSE1+(-0.00011)*H2AFZ+(-0.00025)*ISG20L2+(-0.00001)*KDM1A+(0.00056)*KIAA1841+(0.00071)*KIF20A+(0.00003)*KPNA2+(0)*MARCKS+(0.00037)*MAST2+(0.00021)*MEX3A+(0.00043)*MRPL9+(0.00001)*NAP1L1+(0.00031)*NDC80+(0.00005)*NUP43+(-0.00019)*PAK1IP1+(0.00079)*PDSS1+(-0.00032)*PRPF3+(0.00011)*PSMD1+(0.00013)*PYGO2+(-0.00245)*RAD54B+(0.00001)*RBM17+(-0.00025)*RBM28+(0.00003)*SLC39A1+(-0.00061)*SMYD5+(-0.00001)*SPATS2+(0.00004)*SRSF2+(-0.0003)*SSB+(0.00025)*TCOF1+(0.00036)*TTK+(-0.00007)*UBE2E1+(-0.00013)*WDR75+(-0.00166)*ZFP69B+(-0.00019)*ZNF207
